# Nitrogen Fertilisation Modulates Photosynthetic Performance and Antioxidant Defence Mechanisms in Intercropped Cactus Under Semi-Arid Conditions

**DOI:** 10.3390/plants14243841

**Published:** 2025-12-17

**Authors:** Lady Daiane Costa de Sousa Martins, Alexandre Maniçoba da Rosa Ferraz Jardim, Wagner Martins dos Santos, José Edson Florentino de Morais, Luciana Sandra Bastos de Souza, Lara Rosa de Lima e Silva, Pedro Paulo Santos de Souza, Agda Raiany Mota dos Santos, Wilma Roberta dos Santos, Cleber Pereira Alves, Elania Freire da Silva, Hugo Rafael Bentzen Santos, Carlos André Alves de Souza, José Francisco da Cruz Neto, Adriano Nascimento Simões, Sérgio Luiz Ferreira-Silva, Jiaoyue Wang, Xuguang Tang, João L. M. P. de Lima, Thieres George Freire da Silva

**Affiliations:** 1 Department of Agricultural Engineering, Federal Rural University of Pernambuco, Recife 52171-900, PE, Brazil; ladydaianecsm@gmail.com (L.D.C.d.S.M.); wagnnerms97@gmail.com (W.M.d.S.); joseedson50@gmail.com (J.E.F.d.M.); agdaraiany8@gmail.com (A.R.M.d.S.); carlosandre08_@msn.com (C.A.A.d.S.); thieres.silva@ufrpe.br (T.G.F.d.S.); 2 Institute of Remote Sensing and Geosciences, Hangzhou Normal University, Hangzhou 311121, China; xgtang@hznu.edu.cn; 3 Department of Biodiversity, Institute of Biosciences, São Paulo State University—UNESP, Rio Claro 13506-900, SP, Brazil; 4 Academic Unit of Serra Talhada, Federal Rural University of Pernambuco, Serra Talhada 56909-535, PE, Brazil; luciana.sandra@ufrpe.br (L.S.B.d.S.); lara.rosa@ufrpe.br (L.R.d.L.e.S.); pedro.paulossouza057@gmail.com (P.P.S.d.S.); cleberp.agro@gmail.com (C.P.A.); hugobentzen@hotmail.com (H.R.B.S.); adriano.simoes@ufrpe.br (A.N.S.); sergio.luiz@ufrpe.br (S.L.F.-S.); 5 Department of Botany, Federal University of Pernambuco, Recife 50670-901, PE, Brazil; wilma.roberta@ufpe.br; 6 Department of Agricultural and Forestry Sciences, Federal Rural University of the Semi-Arid, Mossoró 59625-900, RN, Brazil; elania.silva@alunos.ufersa.edu.br; 7 Department of Agronomy, State University of Maranhão, Balsas 65800-000, MA, Brazil; zenetto.agronomia@gmail.com; 8 Institute of Applied Ecology, Chinese Academy of Sciences, Shenyang 110016, China; wangjiaoyue@iae.ac.cn; 9 MARE—Marine and Environmental Sciences Centre, ARNET—Aquatic Research Network, Department of Civil Engineering, Faculty of Sciences and Technology, University of Coimbra, Rua Luís Reis Santos, Pólo II—Universidade de Coimbra, 3030-788 Coimbra, Portugal

**Keywords:** CAM photosynthesis, gas exchange, intercropping systems, photosynthesis, productivity

## Abstract

Agriculture in semi-arid regions faces challenges, such as water scarcity and low soil fertility, making the forage cactus a highly important crop due to its crassulacean acid metabolism (CAM) pathway. The productivity of the forage cactus, however, depends on proper water and nutrient management, especially nitrogen. Despite its importance, there is little research into the effects of nitrogen fertilisation on productive, photochemical, physiological and biochemical parameters, or on intercropping systems. Increasing doses of nitrogen are assumed to enhance CAM pathway, improving productivity, gas exchange, photochemical efficiency and antioxidant accumulation, in addition to mitigating the effects of oxidative stress under adverse conditions. The experiment was conducted in Serra Talhada, Pernambuco, Brazil, in a randomised block design with four replications. Changes in the biometric, productive, photochemical, physiological and biochemical parameters were evaluated in forage cactus intercropped with sorghum (*Sorghum bicolor*) or pigeon pea (*Cajanus cajan*) subjected to different doses of nitrogen (0, 75, 150, 300 and 450 kg ha^−1^). The results showed that nitrogen fertilisation promoted a higher photosynthetic rate and greater stomatal conductance, increased transpiration, and higher levels of pigment and soluble proteins, in addition to reducing lipid peroxidation. Our findings revealed that the cactus—pigeon pea intercropping system has better photosynthetic, enzymatic and productive performance at a dose of 150 kg N ha^−1^, whereas the cactus—sorghum intercropping system required 450 kg N ha^−1^ to achieve similar results. Overall, proper nitrogen management in intercropping systems can optimise the physiological performance and productivity of the forage cactus in semi-arid environments.

## 1. Introduction

Economic and environmental sustainability are pillars of modern agriculture, albeit under pressure from the rapid growth of the global population and from climate change, which affects crops due to water scarcity, rising temperatures and more frequent droughts [1]. These challenges are particularly critical in semi-arid regions, where plants are frequently exposed to combined stresses arising from drought, water and soil salinity, high temperatures and intense solar radiation, all of which directly impact crop growth and productivity [2]. These constraints highlight the urgent need to produce more food through the sustainable use of such inputs as water and fertiliser. The solution may lie in understanding plant photosynthesis and water use efficiency—WUE [3]. Agricultural productivity is directly linked to the accumulation of plant biomass, which depends on photosynthetic efficiency. The products resulting from this process are the main source of biomass in agricultural crops [1].

Crassulacean acid metabolism (CAM) is a physiological modification of plants in arid regions that protects them from the harmful effects of drought. Opening the stomata at night enables carbon dioxide (CO_2_) fixation, reducing water loss through transpiration [2]. As a result, their water use efficiency can be up to six times higher than that of C3 plants and three times higher than that of C4 plants under comparable conditions, making the CAM pathway one of the most effective strategies for coping with water scarcity [4]. Furthermore, during the dry season, plants with CAM pathway alter their physiological responses by accumulating osmoregulators, changing photochemical processes, adjusting the levels of photosynthetic pigments and modifying the synthesis of macromolecules and antioxidants [5].

The forage cactus plays a crucial role in supplying forage, particularly in semi-arid regions, due to its CAM pathway, which affords high resistance to abiotic stress [2,6]. However, to achieve maximum productivity, the crop requires proper water and nutrient management [7]. Nitrogen fertilisation is widely used to increase crop productivity [8] since nitrogen is an essential element for plant growth and development, acting as a constituent of amino acids, proteins, nucleic acids and the cell wall [1,9].

In recent years, several studies have highlighted the role of nitrogen fertilisation in forage cactus. Dantas Neto et al. [7], who investigated growth and productivity in the forage cactus (*Opuntia ficus-indica*) under micro-irrigation and nitrogen fertilisation, found that applying nitrogen afforded an increase in growth, green biomass production, water use efficiency and economic water productivity. Similarly, Santos et al. [8], evaluating the effect of four levels of nitrogen on the morphophysiological, productive, nutritional and economic response of the ‘Orelha de Elefante Mexicana’ clone of the forage cactus [*Opuntia stricta* (Haw.) Haw.] under irrigated conditions in the semi-arid region, found that doses of 300 kg N ha^−1^ or more increased fresh and dry matter productivity in addition to increasing the nutrient content. Silva et al. [10] found increased productivity and water use efficiency in an intercropping system of forage cactus and sorghum fertilised with 150 kg N ha^−1^, showing the beneficial effects of nitrogen fertilisation on forage cactus development. The responses observed in forage cactus studies can vary widely, from substantial increases in productivity to minimal effect. Such inconsistencies are frequently linked to site-specific factors, including differences in initial soil fertility, local water availability, and the nutrient use efficiency of the clones employed, as well as variations in planting density, arrangement, and management practices [11,12,13,14]. In addition to these agronomic responses, effective nitrogen management can also mitigate drought-induced damage by sustaining physiological regulation and enhancing the detoxification of reactive oxygen species (ROS), thereby reducing oxidative stress under water limitation [15]. However, there are still few studies reporting the effects of nitrogen fertilisation on the photochemical and physiological parameters of plants with CAM pathway, or the effect of nitrogen on biochemical parameters, such as the levels of photosynthetic pigments, osmoprotectants and markers of ROS, or the accumulation of antioxidants.

Semi-arid regions present high seasonality and interannual climate variability, with high temperatures, low rainfall, irregular rainfall distribution over space and time, was well as high water and soil salinity [6]. In these environments, plants often face stressful conditions, which causes an imbalance between light capture and its use in CO_2_ assimilation [2]. This excess energy overloads the thylakoids and leads to the formation of ROS, whose accumulation in the chloroplasts causes damage to the photosynthetic apparatus, primarily affecting PSII, and resulting in photoinhibition [16]. To protect themselves under these conditions, plants activate defence mechanisms, synthesising antioxidant compounds that help neutralise the ROS [17,18].

Growing the forage cactus in an intercropping system is a viable alternative for increasing production and the efficient use of available resources in semi-arid regions [10,19]. An increase in productivity, economic return and water use efficiency was seen when using cactus–sorghum [10] and cactus–millet [6] intercropping systems; however, a cactus–pigeon pea system did not show similar behaviour [20]. The effectiveness of intercropping systems varies according to the species and type of photosynthetic metabolism (C3, C4 or CAM), with possible competition for nutrients, water and space, which leads to loss of production and oxidative stress [10,21]. In these systems, the diversification of root architectures promotes a more comprehensive exploration of the soil profile, enhancing the absorption of water and nutrients [22,23]. Additionally, the plant association can modify the microclimate by attenuating solar radiation and reducing evapotranspiration losses, contributing to improved physiological performance [22,23]. Alves et al. [21], investigating regulation of the antioxidant defence system in three forage cactus genotypes grown in an intercropping system, found that, compared to the single crops, intercropping increased the levels of photosynthetic pigments, superoxide dismutase (SOD) and catalase (CAT) enzyme activity, as well as reduced the malondialdehyde (MDA) content. Despite the relevance of forage cactus intercropping, little is known about how nitrogen availability modulates photochemical, biochemical and antioxidant responses in these systems, particularly under brackish-water irrigation.

Our hypothesis is that increased nitrogen availability improves gas exchange, photochemical efficiency, and photosynthetic pigment levels, while simultaneously strengthening antioxidant metabolism and osmotic adjustment, potentially reducing oxidative damage and improving forage cactus productivity in intercropping systems under semi-arid conditions. We also expect the magnitude of these responses to differ between the forage cactus systems with pigeon pea (C3) and sorghum (C4) due to their distinct nitrogen dynamics and competitive capabilities. The aim of this study was to determine how five nitrogen doses influence gas exchange, photochemical performance, antioxidant metabolism, and productivity of the ‘Orelha de Elefante Mexicana’ forage cactus intercropped with sorghum or pigeon pea under brackish water irrigation in the semi-arid region of Brazil.

## 2. Results

### 2.1. The Effect of Nitrogen Fertilisation on Growth and Productivity

Plant height (PH); length of the first-order (CL1), second-order (CL2) and third-order (CL3) cladodes and mean cladode length (CLM); width of the first-order (CW1), second-order (CW2) and third-order (CW3) cladodes and mean cladode width (CWM); thickness of the first-order (CT1), second-order (CT2) and third-order (CT3) cladodes and mean cladode thickness (CTM); perimeter of the second-order (CP2) and third-order (CP3) cladodes and mean cladode perimeter (PCM); area of the first-order (CA1), second-order (CA2) and third-order (CA3) cladodes and mean cladode area (MCA), as well as the dry matter content (DMC) were not significantly affected (*p* > 0.05) by the levels of nitrogen fertiliser (0, 75, 150, 300 and 450 kg ha^−1^) in either of the systems under evaluation (forage cactus intercropped with pigeon pea and forage cactus intercropped with sorghum) (Table 1).

In addition, the perimeter of the first-order cladodes (CP1) in the system with sorghum, and plant width (PW) in the system with pigeon pea showed no significant differences for the levels of nitrogen fertiliser (*p* > 0.05) (Table 1). However, plant width (PW) presented a significant negative linear regression model in the cactus–sorghum intercropping system (*p* < 0.01), showing that the increase in soil nitrogen availability resulted in a 19.7% reduction in plant width (Table 1). On the other hand, in the cactus–pigeon pea system, the perimeter of the first-order cladodes (CP1) was fitted to a significant linear regression model (*p* ≤ 0.05), where increased soil nitrogen content led to a 4% increase in cladode perimeter (Table 1).

**Figure 1:**
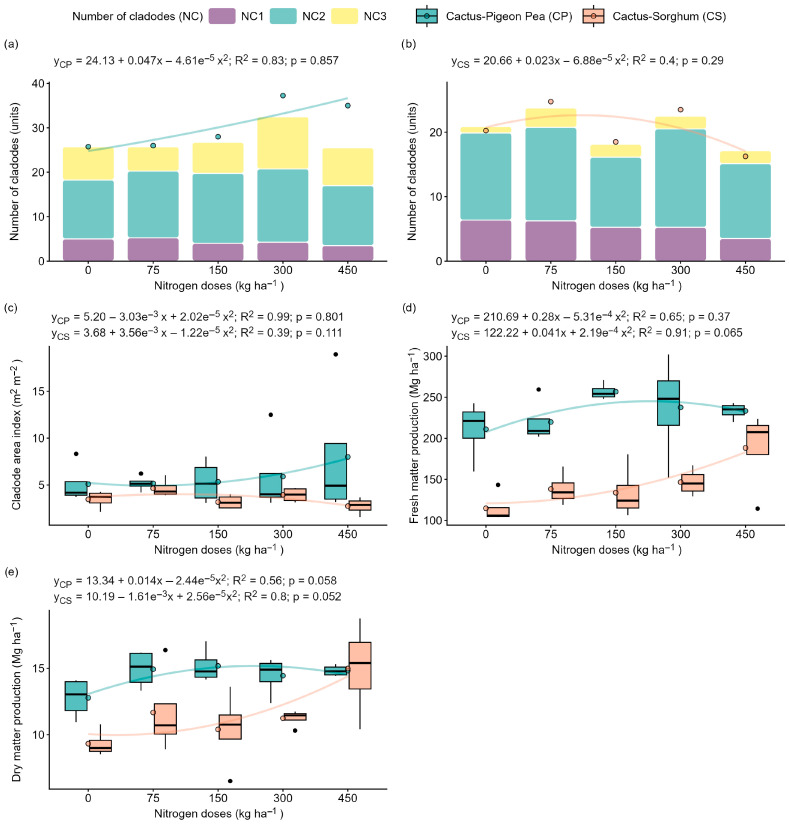
Growth and productivity in the ‘Orelha de Elefante Mexicana’ forage cactus in intercropping systems under different doses of nitrogen fertiliser (0, 75, 150, 300 and 450 kg ha^−1^). Number of first-order (NC1), second-order (NC2) and third-order (NC3) cladodes and total number of cladodes in forage cactus intercropped with pigeon pea (**a**) and with sorghum (**b**), cladode area index (**c**), fresh matter (**d**) and dry matter (**e**) productivity. Each box represents the interquartile range (25th–75th percentile), with whiskers extending to the 5th and 95th percentile values, and a horizontal line within the box indicating the median. The solid lines represent the regression fits of the dependent variables versus nitrogen doses.

The number of first-order (NC1), second-order (NC2) and third-order (NC3) cladodes, the total number of cladodes (TNC), and the cladode area index were not significantly influenced (*p* > 0.05) by the levels of nitrogen fertiliser (0, 75, 150, 300 and 450 kg ha^−1^) in either of the systems under evaluation: forage cactus intercropped with pigeon pea and forage cactus intercropped with sorghum (Figure 1a,b). There was increased development of second-order (NC2) and third-order (NC3) cladodes in the cactus–pigeon pea system, while the cactus–sorghum system favoured the development of first-order (NC1) and second-order (NC2) cladodes (Figure 1a,b).

In both the cactus–pigeon pea and cactus–sorghum systems, fresh matter production and cladode area index (CAI) did not show significant responses to nitrogen fertilisation (*p* > 0.05) (Figure 1c,d). In the cactus cultivation system with pigeon pea and sorghum, dry matter productivity fit significant quadratic regression models (*p* ≤ 0.05). Increasing the nitrogen dose from 0 to 300 kg ha^−1^ increased dry matter production in the pigeon pea system by 24%; however, there was a loss of productivity at 450 kg N ha^−1^ (Figure 1e). In turn, the cactus–sorghum, a dose of 450 kg N ha^−1^ provided a significant increase in dry matter production (87%) compared to a dose of 0 kg N ha^−1^ (Figure 1e).

### 2.2. Effects of Nitrogen Doses and Cultivation Systems on Chlorophyll a Fluorescence

**Figure 2:**
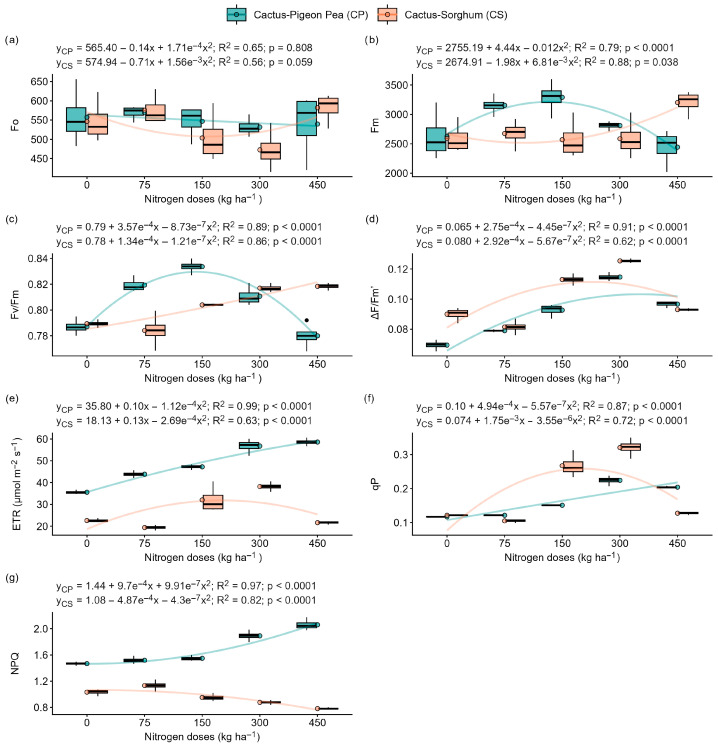
Photochemical responses of the ‘Orelha de Elefante Mexicana’ forage cactus in intercropping systems under different doses of nitrogen fertiliser (0, 75, 150, 300 and 450 kg ha^−1^). Initial fluorescence (Fo) (**a**), maximum fluorescence (Fm) (**b**), maximum quantum yield of photosystem II (Fv/Fm) (**c**), effective quantum yield of photosystem II (ΔF/Fm′) (**d**), relative electron transport rate (ETR, μmol m^−2^ s^−1^) (**e**), photochemical quenching (qP) (**f**) and non-photochemical quenching (NPQ) (**g**). Each box represents the interquartile range (25th–75th percentile), with whiskers extending to the 5th and 95th percentile values, and a horizontal line within the box indicating the median. The solid lines represent the regression fits of the dependent variables versus nitrogen doses.

The initial fluorescence (Fo) was not significantly affected by the levels of nitrogen fertiliser (0, 75, 150, 300 and 450 kg ha^−1^) in either of the systems under evaluation (forage cactus intercropped with pigeon pea and forage cactus intercropped with sorghum) (Figure 2a) (*p* > 0.05). In contrast, the maximum fluorescence (Fm), maximum quantum yield of photosystem II (Fv/Fm), effective quantum yield of photosystem II (ΔF/Fm′), relative electron transport rate (ETR), photochemical quenching (qP) and non-photochemical quenching (NPQ) fit significant quadratic regression models (*p* < 0.0001) in both cropping systems (Figure 2b,c).

In the cactus–pigeon pea system, increasing nitrogen availability from 0 to 150 kg ha^−1^ resulted in increases of approximately 25% in Fm and 6% in Fv/Fm. However, there was a reduction in the values of these parameters at doses of 300 and 450 kg N ha^−1^ (Figure 2b,c). In turn, in the cactus–sorghum system, the highest values for Fm and Fv/Fm were recorded in plants fertilised with 450 kg N ha^−1^, increasing by approximately 24% and 5%, respectively, compared to plants that received no nitrogen fertilisation (0 kg N ha^−1^) (Figure 2b,c). Within this study, in the cactus–pigeon pea system, the highest values for ΔF/Fm′ and qP were seen at a dose of 300 kg N ha^−1^, with increases of 65% and 92%, respectively, compared to a dose of 0 kg N ha^−1^. ETR and NPQ reached their maximum value in plants fertilised with 450 kg N ha^−1^, showing increases of around 65% and 40%, respectively, compared to the lack of any fertiliser (Figure 2d–g). In the cactus–sorghum system, we found that the increase in nitrogen availability from 0 to 300 kg ha^−1^ promoted increases of 39%, 69% and 165% in the values of ΔF/Fm′, ETR and qP, respectively, and a reduction of 15% in the values of NPQ (Figure 2d–g).

### 2.3. Measurement of the Physiological Responses

**Figure 3:**
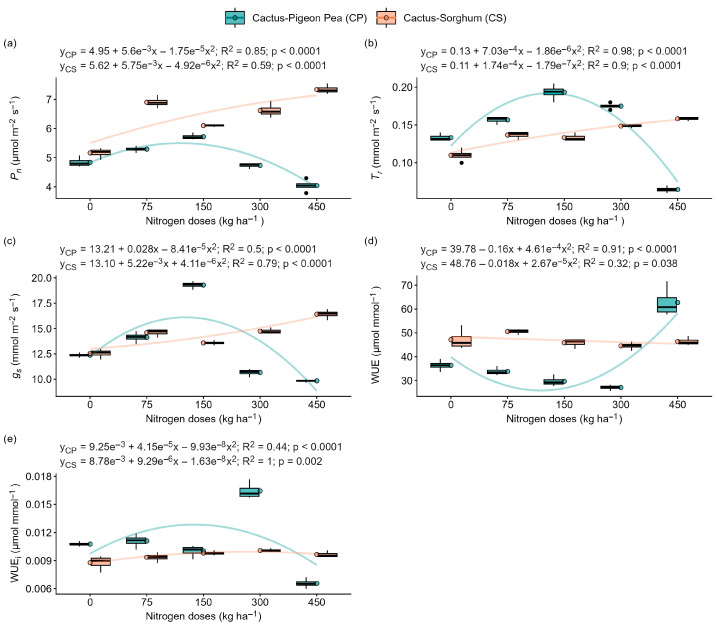
Physiological responses of the ‘Orelha de Elefante Mexicana’ forage cactus in intercropping systems under different doses of nitrogen fertiliser (0, 75, 150, 300 and 450 kg ha^−1^). Net photosynthetic rate (*P_n_*) (**a**), transpiration (*T_r_*) (**b**), stomatal conductance (*g_s_*) (**c**), instantaneous water use efficiency (WUE) (**d**) and intrinsic water use efficiency (WUE_i_) (**e**). Each box represents the interquartile range (25th–75th percentile), with whiskers extending to the 5th and 95th percentile values, and a horizontal line within the box indicating the median. The solid lines represent the regression fits of the dependent variables versus nitrogen doses.

The photosynthetic rate (*P_n_*), transpiration (*T_r_*), stomatal conductance (*g_s_*), and instantaneous (WUE) and intrinsic (WUE_i_) water use efficiency showed a significant quadratic relationship (*p* < 0.05) in both cropping systems (forage cactus–pigeon pea and forage cactus–sorghum) (Figure 3). In the forage cactus–pigeon pea system, the highest values for *P_n_*, *T_r_* and *g_s_* were seen in plants fertilised with 150 kg N ha^−1^, resulting in increases of 18%, 45% and 56%, respectively, compared to a dose of 0 kg N ha^−1^ (Figure 3a–c). Furthermore, increasing the nitrogen dose from 0 to 300 kg ha^−1^ resulted in a 52% increase in WUE (Figure 3d). The highest values for WUE_i_ were recorded at the extreme doses of 0 and 450 kg N ha^−1^ (Figure 3e). On the other hand, in the cactus–sorghum system, increasing nitrogen availability from 0 to 450 kg ha^−1^ resulted in increases of 42%, 44%and 31% in *P_n_*, *T_r_* and *g_s_*, respectively (Figure 3a–c). In this system, the highest values for WUE and WUE_i_ were seen in the unfertilised plants, i.e., with 0 kg N ha^−1^ (Figure 3d,e).

### 2.4. Effect of Nitrogen Application and Intercropping on Photosynthetic Pigment Content

**Figure 4:**
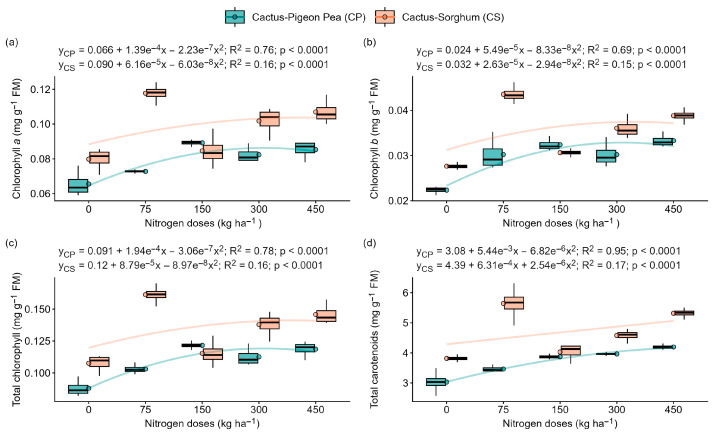
Photosynthetic pigment content of the ‘Orelha de Elefante Mexicana’ forage cactus in intercropping systems under different levels of nitrogen fertiliser (0, 75, 150, 300 and 450 kg ha^−1^). Chlorophyll *a* (**a**), chlorophyll *b* (**b**), total chlorophyll (**c**) and total carotenoids (**d**). Each box represents the interquartile range (25th–75th percentile), with whiskers extending to the 5th and 95th percentile values, and a horizontal line within the box indicating the median. The solid lines represent the regression fits of the dependent variables versus nitrogen doses.

The content of chlorophyll *a*, chlorophyll *b*, total chlorophyll and total carotenoid exhibited a significant fit to a quadratic regression model (*p* < 0.0001) in both cropping systems: forage cactus–pigeon pea and forage cactus–sorghum (Figure 4). In the cactus–pigeon pea system, the levels of chlorophyll *a*, chlorophyll *b*, total chlorophyll, and total carotenoids increased by of 30%, 49%, 38% and 38%, respectively, in response to nitrogen fertiliser application ranging from 0 to 450 kg ha^−1^ (Figure 4a–d). Similarly, in the cactus–sorghum system, pigment levels rose by 34%, 40%, 36% and 39%, respectively, in correlation with increased nitrogen availability (Figure 4a–d).

### 2.5. Lipid Peroxidation, Enzyme Activity and Protein and Carbohydrate Content

**Figure 5:**
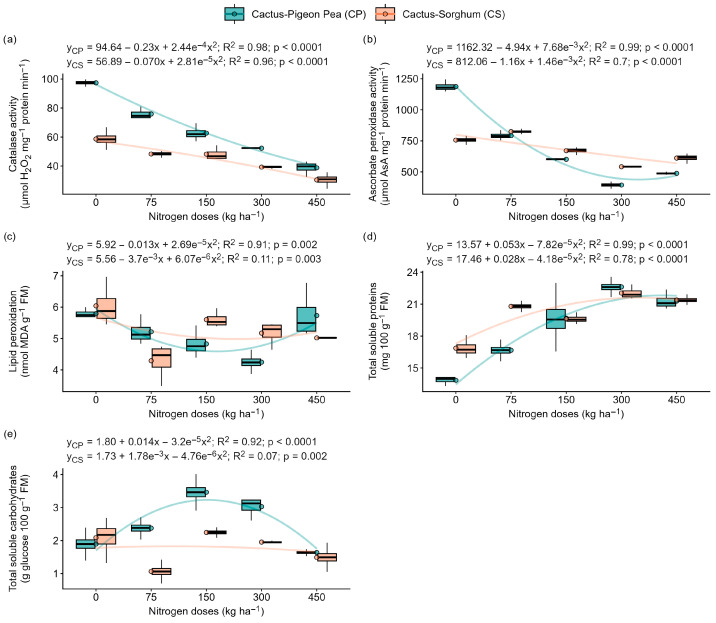
Markers of membrane damage, and the accumulation of antioxidant enzymes, proteins and carbohydrates in the ‘Orelha de Elefante Mexicana’ forage cactus in intercropping systems under different levels of nitrogen fertiliser (0, 75, 150, 300 and 450 kg ha^−1^). Catalase activity (**a**), ascorbate peroxidase activity (**b**), lipid peroxidation (**c**), total soluble proteins (**d**) and total soluble carbohydrates (**e**). Each box represents the interquartile range (25th–75th percentile), with whiskers extending to the 5th and 95th percentile values, and a horizontal line within the box indicating the median. The solid lines represent the regression fits of the dependent variables versus nitrogen doses.

Our results showed that catalase (CAT) and ascorbate peroxidase (APX) activity, lipid peroxidation (MDA), and protein and total soluble carbohydrate contents exhibited significant quadratic responses to nitrogen doses (*p* < 0.05) in both cropping systems (forage cactus–pigeon pea and forage cactus–sorghum) (Figure 5). In the cactus–pigeon pea intercropping system, plants grown without nitrogen fertilisation (0 kg N ha^−1^) showed the highest CAT and APX activity. As nitrogen availability increased from 0 to 450 kg N ha^−1^, CAT and APX activity decreased sharply by 151% and 143%, respectively, relative to the unfertilized control (Figure 5a,b). Conversely, total soluble protein content increased by 63%, reaching maximum values at 300 kg N ha^−1^ (Figure 5d). In contrast, MDA content increased, and total soluble carbohydrate content decreased at the extreme nitrogen levels (0 and 450 kg N ha^−1^) (Figure 5c,e).

In the cactus–sorghum intercropping system, plants grown without nitrogen fertiliser (0 kg N ha^−1^) exhibited the highest CAT activity, MDA content, and total soluble carbohydrate levels. Relative to this control, increasing the nitrogen dose to 450 kg ha^−1^ reduced CAT activity by 48%, decreased MDA content by 17%, and lowered total soluble carbohydrates by 29% (Figure 5a,c,e). In contrast, APX activity increased, reaching its maximum at 75 kg N ha^−1^, while total soluble protein content rose by 26% as nitrogen availability increased from 0 to 300 kg N ha^−1^ (Figure 5d).

### 2.6. Association Between Variables Using Principal Component Analysis

**Figure 6:**
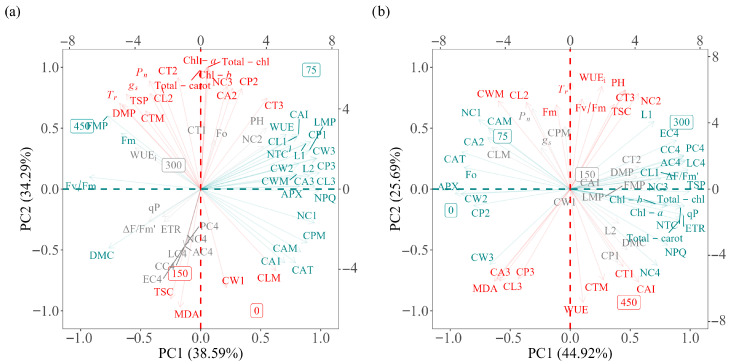
Principal component (PC) analysis of the photochemical, physiological and biochemical variables of the ‘Orelha de Elefante Mexicana’ forage cactus under different levels of nitrogen fertiliser (0, 75, 150, 300 and 450 kg ha^−1^) intercropped with sorghum (**a**) and pigeon pea (**b**). The PCA was performed using mean values of all analysed variables. Variables in green are allocated to the first component (PC1), in red to the second component (PC2) in grey to components three and four (PC3 and PC4).

In the cactus–sorghum intercropping system, principal component analysis (PCA) explained 72.88% of the total variance in the data resulting from the application of different doses of nitrogen (0, 75, 150, 300 and 450 kg ha^−1^), distributed between two principal components: PC1 (38.59%) and PC2 (34.29%) (Figure 6a). The fresh (FMP) and dry matter production (DMC), maximum fluorescence (Fm) and the Fv/Fm ratio showed a positive correlation with each other and with the dose of 450 kg N ha^−1^ (Figure 6a). These parameters, in turn, showed a negative correlation with antioxidant enzyme activity (CAT and APX), the cladode area index (CAI), the non-photochemical extinction coefficient (NPQ), as well as with biometric variables of the first-order cladodes (NC1, CP1, L1, CL1, CA1), second-order (CW2, L2) and third-order cladodes (CW3, CP3, CL3, CA3), in addition to the mean parameters (CPM, CAM, CLM, CWM, LMP) (Figure 6a).

Additionally, the photosynthetic pigment content (chlorophyll-*a*, chlorophyll-*b*, total chlorophyll and total carotenoids), photosynthetic rate (*P_n_*), transpiration (*T_r_*), stomatal conductance (*g_s_*), total soluble proteins (TSP), plant height (PH), the average fresh matter content (DMP) and the development of second-order cladodes (CP2, CA2, CL2, CT2) and third-order cladodes (CT3, NC3), as well as the average cladode thickness (CTM), showed a positive correlation with each other (Figure 6a). These parameters, in turn, were negatively correlated with carbohydrate content (TSC) and MDA, CW1 and CLM levels, which, in turn, showed a positive correlation with each other and with the lowest nitrogen fertiliser doses (0 and 75 kg N ha^−1^) (Figure 6a).

In the cactus–pigeon pea intercropping system, principal component analysis (PCA) explained 70.61% of the total variance of the data as a function of nitrogen dose (0, 75, 150, 300 and 450 kg N ha^−1^), distributed between two principal components: PC1 (45.86%) and PC2 (24.46%) (Figure 6b). Transpiration rate (*T_r_*), soluble carbohydrate content (TSC), maximum fluorescence (Fm), the Fv/Fm ratio, NC2, CT3, CWM, CL2, plant height (PH) and WUE_i_ showed a positive correlation with each other. These parameters showed a negative correlation with MDA content, WUE, the development of first-order cladodes (CT1), second-order (L2) and third-order (CA3, CP3, CL3), as well as with the average cladode thickness (CTM) and the cladode area index (CAI), which, in turn, showed a positive correlation with the 450 kg N ha^−1^ dose (Figure 6b). On the other hand, enzyme activity (CAT and APX), initial fluorescence (Fo), NC1, CAM, CW2, CP2, CA2 and CW3 showed a positive correlation with each other and with the lowest nitrogen doses (0 and 75 kg N ha^−1^) (Figure 6b). These parameters showed a negative correlation with photosynthetic pigments (chlorophyll *a*, chlorophyll *b*, total chlorophyll and total carotenoids), ETR, NPQ, ΔF/Fm′, soluble protein content (TSP), and the development of first-order (CL1, L1), third-order (NC3) and fourth-order cladodes (NC4, EC4, LC4, PC4, AC4, CC4), in addition to the total number of cladodes (NCT), all of which showed a positive correlation with the 300 kg N ha^−1^ dose (Figure 6b).

## 3. Discussion

### 3.1. Productive Performance of the Forage Cactus Is Optimised by Moderate Nitrogen Doses in Intercropping Arrangements

In this study, we identified key factors that limit plant growth in semi-arid environments, particularly the low availability of nutrients, which represents one of the main challenges to productivity in forage species. The forage cactus stands out due to its morphophysiological adaptations, such as its CAM pathway, which favour its cultivation under water-deficit conditions [16]. In these environments, intercropping systems involving species with C3 or C4 metabolism are widely used because they promote greater efficiency in the use of water and nutrients [2,23]. The combination of C3, C4 and CAM species optimises water use due to the temporal differences in stomatal opening and gas exchange [2,10].

In this context, we evaluated the effect of nitrogen availability and intercropping systems with pigeon pea (C3) or sorghum (C4) on the productive, physiological, photochemical and biochemical parameters of the forage cactus. It was observed that, in the cactus–pigeon pea intercropping system, the highest fresh and dry matter productivity and the greatest values of photosynthetic rate (*P_n_*), stomatal conductance *(g_s_*) and transpiration (*T_r_*) occurred with 150 and 300 kg N ha^−1^, whereas in the cactus–sorghum system these same parameters performed best with 450 kg N ha^−1^ (Figure 1d,e and Figure 3a–c). These contrasting responses suggest that moderate nitrogen inputs may be sufficient in the presence of a legume associate, whereas higher doses appear to be required when intercropping with a C4 species of greater competitive capacity.

This response may be associated with the potential ability of legumes, such as pigeon pea, to contribute nitrogen to the forage cactus through processes such as root exudation, nodule decomposition and root turnover. Although no direct evidence of nitrogen transfer was measured in this study, this mechanism is widely supported in the literature and may reasonably help explain the improved performance of the cactus–pigeon pea system under lower nitrogen fertiliser doses [19]. In addition, excess nitrogen in the forage cactus has been associated with reduced dry matter accumulation and decreased nutrient use efficiency [10], which could help explain the reduction in productivity observed in plants fertilised with 450 kg N ha^−1^, in the cactus–pigeon pea intercropping system. As a consequence of the increase in photosynthesis, there was a reduction in instantaneous and intrinsic water use efficiency, especially at doses of 150 kg N ha^−1^ (cactus–pigeon pea) and 300 kg N ha^−1^ (cactus–sorghum) (Figure 3d,e).

In the present study, the highest dry matter yields were 15.06 Mg ha^−1^ for the forage cactus intercropped with pigeon pea under 150 and 300 kg N ha^−1^, and 16.85 Mg ha^−1^ for the cactus–sorghum intercropping system. These values were lower than those reported by Santos et al. [8], who obtained approximately 34 Mg ha^−1^ of dry matter in sole cropping fertilised with 450 kg N ha^−1^. This difference may be related to intensified competition for nutrients, water, light and space in intercropping systems, creating less favourable conditions for growth and possibly inducing oxidative stress [21]. In addition, the higher nutrient demand imposed by sorghum may have accentuated competitive pressure, thereby requiring higher nitrogen inputs to sustain cactus performance. However, the adoption of intercropping systems plays a strategic role in building agroecosystems that are more resilient to climate change, as this arrangement can induce robust adaptive responses, including the modulation of genes related to root development, the regulation of nitrogen metabolism, and the strengthening of plant defence mechanisms [24]. Thus, even when absolute yields are lower under intercropping, the ecological and physiological benefits may outweigh the reduction in biomass, particularly in semi-arid environments where stability and resource-use efficiency are priorities [10].

### 3.2. Nitrogen Deficiency Intensifies Photoinhibition and Compromises Photosynthesis

Chlorophyll fluorescence is a sensitive indicator of the performance of the photosynthetic apparatus under environmental stresses, such as high temperature, drought, salinity and nutrient deficiency [2,25]. The ideal Fv/Fm values range between 0.80 and 0.83 [3]. In this study, plants fertilised with 0 kg N ha^−1^ showed values of 0.79 and 0.78, indicating a reduction in photochemical performance (Figure 2c). Furthermore, the ΔF/Fm′ and qP parameters decreased in the treatments with 0 and 450 kg N ha^−1^ (Figure 2d,f). ΔF/Fm′ represents the proportion of electrons effectively used during the photochemical phase, while qP reflects the fraction of PSII reaction centres that are open and capable of electron transport [16,26].

Nitrogen directly affects the photosynthetic apparatus, regulating Rubisco (ribulose-1,5-bisphosphate carboxylase/oxygenase) content, chlorophyll synthesis, and stomatal conductance [15]. Adequate N availability tends to increase Rubisco accumulation, stimulate chlorophyll biosynthesis and support higher stomatal conductance, collectively improving CO_2_ diffusion and light capture. Thus, reductions in photochemical efficiency under insufficient or excessive N may be related to impairments in these structural and functional components of photosynthesis [15].

These results indicate that low nitrogen availability is likely to compromises the efficient use of electrons for carbon fixation, leading to photoinhibition and damage to PSII, as also evidenced by the decrease in electron transport rate (ETR) and the increase in non-photochemical quenching (NPQ) (Figure 2e,g). The reduction in ETR may be associated with instability in the light-harvesting complexes [26], while the higher NPQ suggests the activation of photoprotective mechanisms, such as the xanthophyll cycle [2].

Nitrogen limitation also affected CAM, resulting in lower values of *P_n_*, *g_s_* and *T_r_* (Figure 3a–c). Under stress conditions, the reduction in stomatal opening decreases CO_2_ assimilation and, consequently, the photosynthetic rate [27]. This imbalance leads to insufficient regeneration of NADP^+^ and the accumulation of NADPH_2_, favouring the diversion of electrons to molecular oxygen and the formation of reactive oxygen species (ROS), such as superoxide anions (O_2_^•−^), hydroxyl radicals (^•^OH) and hydrogen peroxide (H_2_O_2_) [28].

The increase in ROS concentration was confirmed by the higher levels of malondialdehyde (MDA) in the treatments with 0 and 450 kg N ha^−1^ (Figure 5c). The CAM pathway can intensify this ROS generation, as electron transport continues even with the stomata closed, increasing the internal O_2_ concentration [21,29]. As an additional effect, photooxidation and a reduction in photosynthetic pigments were observed in plants with 0 kg N ha^−1^, whereas increased nitrogen availability elevated the levels of chlorophyll *a*, *b*, total chlorophyll and carotenoids (Figure 4a–d), confirming the structural role of nitrogen in the chlorophyll molecule [30]. The findings of this study are consistent with those reported by Chen et al. [18], who observed that nitrogen limitation in *Oryza sativa* triggers a reduction in photosynthetic activity, an increase in Fo, accumulation of H_2_O_2_, and a pronounced activation of the antioxidant system, confirming that nitrogen is essential for maintaining photochemical stability and redox balance under stress.

### 3.3. Nitrogen Supply Strengthens the Antioxidant System and Reduces Oxidative Damage

Under stress conditions, plants adjust their metabolism by modifying the synthesis and concentration of metabolic compounds [31]. In the present study, plants under 0 kg N ha^−1^ showed higher activity of the antioxidant enzymes CAT and APX (Figure 5a,b), which are responsible for detoxifying H_2_O_2_ into H_2_O and O_2_ [31]. The increase in this activity indicates the plant’s attempt to mitigate the damage caused by excess ROS [32]. In environments with resource competition, such as in intercropping systems, this activation may be intensified [21].

Similar results were reported by Chen et al. [18], who demonstrated that nitrogen deficiency significantly increases H_2_O_2_ levels and stimulates the activity of antioxidant enzymes such as SOD, CAT and APX, indicating that nitrogen limitation intensifies oxidative stress and requires greater activation of the antioxidant apparatus.

An increase in soluble protein content was also observed with the increment of nitrogen doses (150, 300 and 450 kg ha^−1^) (Figure 5d), reflecting the role of nitrogen as a structural constituent of proteins [1,33]. On the other hand, soluble carbohydrates decreased with increasing nitrogen, possibly due to the redirection of carbon towards protein synthesis [34]. In the treatment with 0 kg N ha^−1^ in the cactus–sorghum intercropping system, the higher accumulation of carbohydrates may be related to osmoprotective mechanisms under stress and increased competition for resources (Figure 5e).

### 3.4. Multivariate Integration of Productive and Physiological Parameters

The PCA results jointly integrated the productive, physiological, photochemical, and biochemical responses. In the cactus–pigeon pea system, the distribution of variables suggested that moderate nitrogen inputs (150 kg N ha^−1^) likely favoured higher *P_n_*, *g_s_*, fresh and dry matter yield, and cladode area index, possibly due to the legume’s contribution to the nitrogen cycle, reducing the cactus’s dependence on external N inputs [19]. This pattern aligns with evidence that adequate N availability tends to increase Rubisco content, stimulate chlorophyll biosynthesis, and support higher stomatal conductance [15], which typically enhances light capture, CO_2_ diffusion, and photosynthetic efficiency.

In the cactus–sorghum system, the PCA grouped the 450 kg N ha^−1^ treatment with variables associated with photochemical stability (Fv/Fm, Fm) and productivity, indicating that the high competitive capacity of the C4 species [2,20] likely increased nutrient demand, requiring greater N inputs to sustain photosynthetic performance. This outcome is consistent with the observed reduction in ETR and increase in NPQ under N deficiency or excess, which may reflect adjustments in the xanthophyll cycle and energy dissipation mechanisms [2,26].

In both systems, the 0 kg N ha^−1^ treatment clustered with markers of oxidative stress, including higher MDA levels and elevated CAT and APX activity, suggesting that N limitation may have reduced electron flow, restricted CO_2_ assimilation, and increased ROS production in CAM species [21,28,29]. These patterns support the hypothesis that limited N availability compromises chlorophyll content, tends to reduce photochemical efficiency, may destabilise light-harvesting complexes, and stimulates the activation of antioxidant defences [15,26,30].

Overall, the results of this work support our hypotheses by showing that increased nitrogen availability improves the physiological, photochemical, and productive attributes of forage cactus. However, the magnitude of these responses varies between intercropping systems. In the cactus–pigeon pea system, moderate nitrogen supply appears sufficient to improve photosynthesis, pigment accumulation, and biomass production. In the cactus–sorghum system, higher nitrogen doses are permitted to maintain photochemical stability and productivity, reflecting the higher competitive demand of the C4 species. In both systems, nitrogen deficiency is associated with increased markers of oxidative stress and reduced photochemical efficiency, while adequate nitrogen availability attenuates ROS formation and preserves CAM pathway functionality under semi-arid conditions.

## 4. Materials and Methods

### 4.1. Characterisation of the Study Area

**Figure 7:**
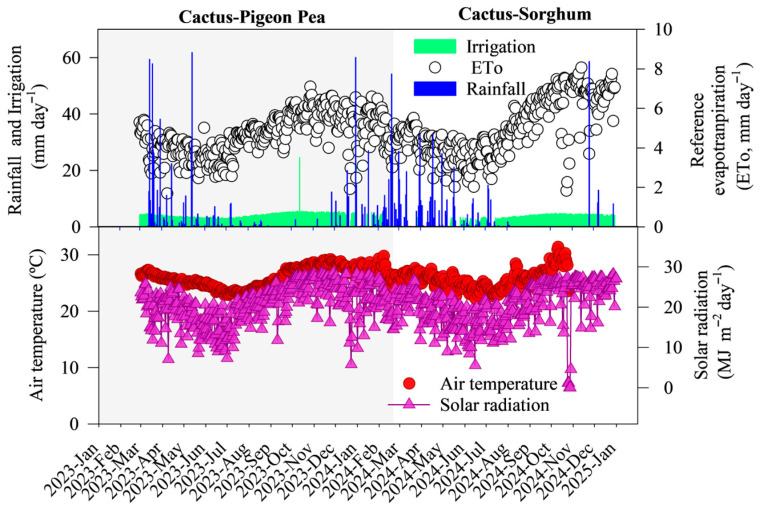
Rainfall, irrigation, reference evapotranspiration (ETo), air temperature and solar radiation during the 2023–2024 experimental period in Serra Talhada, Pernambuco, Brazil. The light grey shading represents the cactus–pigeon pea experiment, and the white shading represents the cactus–sorghum experiment. All meteorological data were obtained from the automatic weather station of the National Institute of Meteorology (INMET), located adjacent to the experimental area.

The experiment was conducted at the International Reference Centre for Agrometeorological Studies of the Cactus and Other Forage Plants at the Serra Talhada Academic Unit of the Federal Rural University of Pernambuco, Serra Talhada, Pernambuco, Brazil (7°59′ S, 38°15′ W, altitude 431 m) during the period from March 2023 to November 2024. According to the Köppen classification, the climate in the region is type BShw’ (hot and dry semi-arid), with a rainy season during the summer and dry winters, an average annual rainfall of 667.2 mm concentrated during the warmest months, relative humidity of 62.3%, potential evapotranspiration in excess of 1800 mm per year, and an average temperature of approximately 25.9 °C [8,35]. The weather conditions during the experimental period are shown in Figure 7.

The soil in the experimental area is classified as a typical Eutrophic Ta Haplic Cambisol with the following physical and chemical attributes at a depth of 0–0.20 m: pH (water) of 5.95, electrical conductivity of the saturated soil paste extract (EC) of 0.32 dS m^−1^, P (Mehlich-1) of 168.96 mg dm^−3^, K^+^ of 13.8 cmol_c_ dm^−3^, Na^+^ of 1.09 cmol_c_ dm^−3^, Ca^2+^ of 3.45 cmol_c_ dm^−3^, Mg^2+^ of 1.90 cmol_c_ dm^−3^, H^+^ + Al^3+^ of 0.6 cmol_c_ dm^−3^, sum of bases (SB) of 20.25 cmol_c_ dm^−3^, cation exchange capacity (CEC) of 20.85 cmol_c_ dm^−3^, base saturation (V%) of 97.15%, organic carbon of 4.6 g kg^−1^, organic matter of 7.93 g kg^−1^, sand of 828.6 g kg^−1^, silt of 148.25 g kg^−1^, clay of 23.15 g kg^−1^ and bulk density of 1.45 g cm^−3^. All soil analyses were performed by a laboratory specialising in physicochemical analysis, according to their standard protocols.

### 4.2. Experimental Design, Treatments and Irrigation Management

The experimental area was prepared on 14 October 2018 by ploughing and harrowing. The study evaluated two experiments with the ‘Orelha de Elefante Mexicana’ forage cactus [*Opuntia stricta* (Haw.) Haw.]. The first experiment was conducted from 24 May 2023 to 20 February 2024 and consisted of the cactus intercropped with pigeon pea [*Cajanus cajan* (L.) Millsp.]. Pigeon pea was sown on 28 March 2023, and two harvest cycles (cut and regrowth) were carried out on 18 September 2023 and 19 February 2024. The second experiment was carried out from 21 February to 19 November 2024 and consisted of the forage cactus intercropped with the sorghum cultivar ‘Ponta Negra’. Sorghum was sown on 6 June 2024, and two harvest cycles (cut and regrowth) were performed on 21 August 2024 and 23 October 2024.

In this study, the forage cactus was planted at a spacing of 1.25 m between rows and 0.20 m between plants, giving a density of 40,000 plants per hectare. The intercrops (pigeon pea and sorghum) were spaced at a distance of 0.20 m from the rows of cactus. The cladodes were planted in a dominant alignment with 50% of their lower ends inserted into the soil. The treatments were applied at the start of each cycle, with the addition of 80 kg ha^−1^ phosphorus (P) and 130 kg ha^−1^ potassium (K^+^). Nitrogen (N) was applied in the form of urea in doses of 0, 75, 150, 300 and 450 kg N ha^−1^. The experiment was conducted in a randomised block design (RBD) with three replications, giving a total of 15 plots. Each plot contained four rows of 12 plants and occupied an area of 15 m^2^. The two central rows were considered working plots, except for the two plants at each end. The total experimental area comprised 225 m^2^.

Irrigation was carried out using a drip system with a spacing of 0.2 m between emitters, flow rate of 1.67 L h^−1^ and application uniformity of 90%, operating at a pressure of 1 atm. Irrigation took place on fixed days (Mondays, Wednesdays and Fridays). The irrigation depth during each cycle was 100% of the reference evapotranspiration (ETo), which was calculated using the Penman–Monteith equation, standardised as per FAO Bulletin 56 [36]. The mean irrigation depth applied throughout the experiment was 3.16 mm day^−1^. The water used for irrigation came from an artesian well located at the Serra Talhada Academic Unit–UFRPE, and had an average electrical conductivity of 1.51 dS m^−1^ and a pH of 6.84, being classified as C3 (high salinity) according to the Richards classification [37]. The average sodium and potassium concentrations were 168.66 mg L^−1^ and 28.17 mg L^−1^, respectively [6].

The irrigation depth varied due to the rainfall events that occurred throughout the experimental period. We did not carry out irrigation on rainy days or immediately after rainfall events. The volume of irrigation water, the rainfall and the reference evapotranspiration per growth cycle are shown in Figure 1. Data on air temperature (mean, maximum and minimum, in °C), relative humidity (mean, maximum and minimum, in %), atmospheric pressure (mean, maximum and minimum, in hPa), wind speed (m s^−1^), global solar radiation (MJ m^−2^) and variation in rainfall (mm) were obtained from the automatic weather station of the National Institute of Meteorology (INMET) located next to the experimental area.

### 4.3. Evaluating Growth and Productivity

At the end of each cycle, biometric evaluations were carried out that included plant height (PH, cm), plant width (PW, cm), number of cladodes (NC, units) based on order of emergence, and total number of cladodes (TNC, units). One representative unit from each order was selected to measure cladode length (CL, cm), width (CW, cm), perimeter (CP, cm) and thickness (CT, mm), as per the methodology of Jardim et al. [38]. From these data, the cladode area (CA, cm^2^) and the cladode area index (CAI, m^2^ m^−2^) were calculated using the equations described by Pinheiro et al. [39] and Silva et al. [40]:

(1) $$\mathrm{CA}\;=\;0.7086\;\times \left[\frac{1\;-\exp(-0.000045765\;\times \;\mathrm{CL}\;\times \;CW)}{0.000045765}\right]$$

(2) $$\mathrm{CAI}=\frac{\frac{\sum_{n}^{i=1}\left(\mathrm{CA}\right)}{10,000}}{S1\;\times \;S2}$$

where CA is the cladode area; CL is the cladode length; CW is the cladode width; CAI is the cladode area index; 10,000 is the conversion factor from cm^2^ to m^2^; S1 is the spacing between rows; S2 is the spacing between plants.

Productivity was assessed at the end of each cycle (20 February 2024 and 19 November 2024) by counting the plants within the working plot to determine the final plant density. All the plants were then harvested and weighed to obtain the total fresh weight (kg), keeping only the primary and basal cladodes in the field. To determine the dry matter, two representative cladodes from each plot were selected, weighed, broken up, stored in labelled paper bags and placed in a forced-air oven (Marconi, MA033/1080, Piracicaba, SP, Brazil) at 55 °C to constant weight. For the assay, the dry matter content (DMC) was obtained as the ratio of cladode dry weight to fresh weight. Fresh matter productivity (FM, Mg ha^−1^) was calculated by multiplying the total fresh weight by the final plant density, while dry matter productivity (DM, Mg ha^−1^) was estimated by multiplying the final fresh weight by the dry matter content.

### 4.4. Measurements of Gas Exchange

Measurements were taken on three plants per treatment, in February and August 2024 between 22:00 and 02:00 using a portable infrared gas analyser (CI-340, CID Bio-Science, Camas, WA, USA). The interval was defined based on prolonged preliminary tests in the experimental environment itself, which identified this period as the one in which the plants exhibited the highest photosynthetic activity. The camera was manufactured by CID Bio-Science, and designed specifically for measuring forage cactus cladodes, with an area of 14.70 cm^2^. The study employed an open system, with an air flow rate of 0.30 L min^−1^. During nighttime readings, the photosynthetic photon flux density (PPFD) was zero. In February, an average atmospheric CO_2_ concentration of 449.1 ± 11.3 µmol mol^−1^ was recorded, along with a leaf temperature of 22.1 ± 0.3 °C and an air temperature of 26.8 ± 0.1 °C. In August, the average CO_2_ concentration was 437.0 ± 61.3 µmol mol^−1^, while the leaf and air temperatures were 16.4 ± 0.5 °C and 21.6 ± 0.9 °C, respectively. The following parameters were measured in the secondary cladodes: net photosynthetic rate (*P_n_*, µmol CO_2_ m^−2^ s^−1^), transpiration (*T_r_*, mmol H_2_O m^−2^ s^−1^), stomatal conductance (*g_s_*, mmol m^−2^ s^−1^), instantaneous water use efficiency (WUE = *P_n_*/*T_r_*) and intrinsic water use efficiency (WUE_i_ = *P_n_*/*g_s_*).

### 4.5. Chlorophyll Fluorescence

Chlorophyll *a* fluorescence was measured on three plants per treatment in February and August 2024 between 09:00 and 12:00 using a portable pulse-modulated fluorometer (Mini-PAM II, Heinz Walz, Effeltrich, BY, Germany). Briefly, the measurements were taken on secondary cladodes following a dark-adaptation period of 50 min, using support clips fixed around each cladode [2]. After dark adaptation, the cladodes were exposed to a saturation flash with a photosynthetic photon flux density of approximately 6000 μmol m^−2^ s^−1^ for 10 s, allowing the determination of PSII parameters of initial fluorescence (Fo), maximum fluorescence (Fm), and maximum quantum yield (Fv/Fm). Subsequently, the cladodes were exposed to actinic light with a photosynthetic photon flux density of 1500 μmol m^−2^ s^−1^ for 20 s, enabling the determination of the effective yield of photosystem II (ΔF/Fm′), photochemical quenching (qP), non-photochemical quenching (NPQ), and relative electron transport rate (ETR).

### 4.6. Biochemical Evaluations

Fresh secondary cladodes were collected in the field during February and August 2024, between 09:00 and 10:00 a.m., identified and taken to the laboratory. Samples of epidermis were then taken from the central region of each cladode [2]. The samples were immediately frozen in liquid nitrogen and stored in an ultra-low temperature freezer (−80 °C) for later biochemical analysis, remaining stored for approximately 180 days before processing.

### 4.7. Chlorophyll a and b and Total Carotenoids

In the present study, the levels of chlorophyll *a* (Chl-*a*), chlorophyll *b* (Chl-*b*), total chlorophyll (Chl-total) and total carotenoids (Car-total) were determined using an adapted version of the methodology proposed by Lichtenthaler and Buschmann [41]. Samples of 0.2 g of cladode epidermis were weighed and solubilised in 2.5 mL of 80% acetone. The samples were then centrifuged (Hettich, MIKRO 220 R, Kirchlengern, NW, Germany) at 6000× *g* for 15 min at 4 °C. The supernatant was collected, and readings were taken at wavelengths of 470, 645 and 663 nm with the help of a spectrophotometer (Biochrom, Libra S8, Cambridge, England, UK) with a 1 cm quartz cuvette. The Chl-*a*, Chl-*b*, Chl-total and Car-total content, expressed in mg g^−1^ FM, was calculated using the following equations:

(3) $$\mathrm{Chl}\text{-}a\;=\left[\frac{(12.25{\;\times \;A}_{663}\;-\;2.79{\;\times \;A}_{645})}{1000\;\times \;W}\right]\;\times \;V$$

(4) $$\mathrm{Chl}\text{-}b=\left[\frac{(21.5{\;\times \;A}_{645}-\;5.10{\;\times \;A}_{663})}{1000\;\times \;W}\right]\;\times \;V$$

(5) Chl-total=Chl-a+Chl-b

(6) $$\mathrm{Carotenoids}=\left[\frac{\left(1000\;\times \;A_{470}\right)-\left(1.82\;\times \;\mathrm{Chl}\text{-}a\right)-\left(84.02\;\times \;\mathrm{Chl}\text{-}b\right)}{198}\right]\;$$

where *A* is the absorbance; V is the final volume of the extract (2.5 mL); W is the weight in grams of the plant tissue (0.2 g).

### 4.8. Total Soluble Carbohydrates and Lipid Peroxidation

The total soluble carbohydrates were determined using the methodology proposed by Dubois et al. [42] with modifications. Samples of 0.2 g of cladode epidermis were weighed and homogenised in 1.5 mL of 0.1 M potassium phosphate buffer (pH 7.0). The homogenate was then centrifuged (Hettich, MIKRO 220 R, Kirchlengern, NW, Germany) at 12,000× *g* for 15 min at 4 °C, and 25 μL of the extract was added to test tubes together with 475 μL of ultrapure water, 500 μL of 5% phenol and 2500 μL of sulphuric acid (p.a.). The tubes were then stirred by magnetic stirrer, left to rest for 15 min and kept in an ice bath until readings were taken at 490 nm with the help of a spectrophotometer (Biochrom, Libra S8, Cambridge, England, UK). The calibration curve was generated using an anhydrous glucose solution (0–1 μmol mL^−1^). The total soluble carbohydrate content was expressed in g of carbohydrates per 100 g of fresh matter (FM).

The level of lipid peroxidation was determined from the level of thiobarbituric acid-reactive substances (MDA), using the methodology described by Coelho Júnior et al. [43] with modifications. Samples of 0.1 g of cladode epidermis were homogenised in 1 mL of 6% trichloroacetic acid (TCA). The homogenate was then centrifuged at 7960× *g* for 15 min at 4 °C, and a 0.5 mL aliquot of the supernatant was removed, to which 1.5 mL of a reaction solution containing 0.5% thiobarbituric acid (TBA) in 20% TCA was added. The mixture was heated to 95 °C for 30 min and then cooled in an ice bath for a further five minutes. Readings were taken by spectrophotometer (Biochrom, Libra S8, Cambridge, England, UK) at wavelengths of 532 and 660 nm. The MDA content was calculated using an absorption coefficient of 155 mmol^−1^ cm^−1^ and expressed in nmol g^−1^ of fresh matter (FM).

### 4.9. Enzyme Extraction and Assay

To obtain the enzyme extract, 0.1 g of cladode epidermis was macerated with liquid nitrogen and homogenised in 2 mL of 0.1 M potassium phosphate buffer (pH 7.0) containing 10 mM EDTA, 200 mM of ascorbic acid (AsA) and 1% (*w*/*v*) insoluble polyvinylpolypyrrolidone (PVPP). The extract was then centrifuged (Hettich, MIKRO 220 R, Kirchlengern, NW, Germany) at 12,000× *g* for 23 min at 4 °C.

### 4.10. Total Soluble Proteins (TSP), Catalase (CAT) and Ascorbate Peroxidase (APX) Activity

The total soluble protein content in the enzyme extract was determined as per Bradford [44], with modifications. Aliquots of 10 μL of the supernatant (obtained in Section 4.9) were added to 90 μL of potassium phosphate buffer, pH 7.0 (0.1 M) and 1000 μL of Bradford solution, homogenised on a tube shaker, and allowed to stand for 15 min. Following the incubation period, readings were taken at 595 nm. The protein content was estimated based on a standard curve prepared using bovine serum albumin (BSA), and determined in mg protein g^−1^ FM.

Catalase activity was determined as per Cakmak et al. [45] and Havir and McHale [46] with modifications. For the assay, 5 μL aliquots of the enzyme extract (obtained in Section 4.9) were added to 45 μL of ultrapure water and 900 μL of potassium phosphate buffer (50 mM, pH 7.0) and kept in a water bath at 27 °C. Just before taking the readings, 50 μL of H_2_O_2_ (20 mM) was added. The absorbance decay at 240 nm was monitored for 1.5 min, with readings taken by spectrophotometer every 10 s. Catalase activity was calculated based on a molar extinction coefficient of 39.4 mM^−1^ cm^−1^ for H_2_O_2_, and expressed in μmol H_2_O_2_ mg^−1^ protein min^−1^.

Ascorbate peroxidase (APX) activity was determined as per the methodology proposed by Nakano and Asada [47] with modifications. For the assay, 10 μL aliquots of the enzyme extract (obtained in item 4.9) were added to 940 μL of 50 mM potassium phosphate buffer (pH 6.0) containing 0.5 mM ascorbic acid, and kept in a water bath at 27 °C for five minutes. Before taking the readings, 50 μL of H_2_O_2_ (30 mM) were added. The absorbance decay at 290 nm was monitored for 1.5 min, with readings taken by spectrophotometer every 10 s. Ascorbate peroxidase activity was calculated based on a molar extinction coefficient of 2.8 mM^−1^ cm^−1^, and expressed in μmol AsA mg^−1^ protein min^−1^.

### 4.11. Data Processing and Statistical Analysis

The data were initially assessed for normality using the Shapiro–Wilk test and for homoscedasticity using Levene’s test. They were then submitted to analysis of variance (ANOVA) considering the levels of nitrogen fertiliser (0, 75, 150, 300 and 450 kg ha^−1^) for both experiments (cactus–pigeon pea and cactus–sorghum) as the treatments. Whenever the F-test indicated significance, we fitted both linear and non-linear regression models for the fertilisation levels. Principal component analysis (PCA) was also carried out using the mean values of the analysed variables, decomposing these variables into sets of orthogonal vectors. The R software (v.4.4.2) was used for the statistical analysis and to generate the graphs.

## 5. Conclusions

This study demonstrates that the physiological, biochemical, antioxidant, and productive responses of forage cactus in semi-arid environments are strongly shaped by the interaction between nitrogen availability and intercropping systems. Moderate nitrogen fertilisation (150 kg N ha^−1^) supports improved gas exchange, photochemical stability, and antioxidant activity in the cactus–pigeon pea system, while higher nitrogen supply (450 kg N ha^−1^) is required to sustain comparable functional responses in the cactus–sorghum system. These enhancements in photosynthetic efficiency and oxidative protection likely contribute to increased biomass accumulation and yield, reflecting the cactus’s capacity to adjust its metabolism according to resource availability and competitive pressure within the intercrop.

The results also suggest that combining intercropping with moderate nitrogen fertilisation can maintain forage cactus productivity while reducing reliance on high nitrogen inputs, thereby contributing to more sustainable semi-arid agriculture through improved resource-use efficiency and system resilience.

Although the study demonstrated that the physiological, biochemical, antioxidant, and productive responses of forage cactus are strongly influenced by intercropping and nitrogen availability, some limitations should be considered. The evaluations focused exclusively on the forage cactus, without a detailed assessment of the photochemical, physiological, and biochemical responses of the accompanying species, such as pigeon pea and sorghum. This gap limits a comprehensive understanding of the interspecific interactions that determine the performance of intercropped systems.

Therefore, future research is needed to investigate how nitrogen fertilisation directly affects these associated crops, as well as how such changes modulate competition and complementarity within the intercrop. Incorporating these analyses will help elucidate key ecological and physiological mechanisms and provide more robust support for the integrated management of forage cactus-based production systems in semi-arid regions.

## Figures and Tables

**Table 1:** Effect of different levels of nitrogen fertiliser (0, 75, 150, 300 and 450 kg ha^−1^) on biometric variables in the ‘Orelha de Elefante Mexicana’ forage cactus.

Variable	Experiment	Nitrogen Dose (kg ha^−1^)					Equation	R^2^	*p*-Value
		0	75	150	300	450			
PH (cm)	Cactus–pigeon pea	96.8	99.5	103.5	105.3	94.8	y = 95.70 + 0.084x − 1.88e^−4^x^2^	0.93	0.63
	Cactus–Sorghum	72.0	86.3	80.5	78.8	72.8	y = 75.42 + 0.072x − 79e^−4^x^2^	0.55	0.11
PW (cm)	Cactus–pigeon pea	62.0	64.6	52.6	73.0	68.0	y = 59.88 + 0.021x	0.26	0.75
	Cactus–Sorghum	53.8	62.6	50.0	55.1	44.8	y = 57.80 − 0.023x	0.41	0.00
CL1 (cm)	Cactus–pigeon pea	28.3	27.5	30.8	31.6	29.8	y = 27.42 + 0.026x − 4.42e^−5^x^2^	0.68	0.51
	Cactus–Sorghum	31.3	33.3	31.3	32.1	30.3	y = 31.63 + 7.86e^−3^x − 2.41e^−5^x^2^	0.48	0.90
CL2 (cm)	Cactus–pigeon pea	27.3	31.6	27.3	28.0	25.8	y = 29.21 − 6.35e^−3^x	0.27	0.43
	Cactus–Sorghum	23.8	29.6	27.6	30.1	29.6	y = 24.86 + 0.035x − 5.54e^−5^x^2^	0.68	0.37
CL3 (cm)	Cactus–pigeon pea	29.5	26.5	27.9	23.3	29.0	y = 29.82−0.041x + 8.39e^−5^x^2^	0.61	0.75
	Cactus–Sorghum	18.8	22.6	16.3	16.3	12.3	y = 20.62 − 0.017x	0.68	0.52
CLM (cm)	Cactus–pigeon pea	21.3	21.0	17.5	19.6	18.3	y = 20.63 − 5.65e^−3^x	0.38	0.83
	Cactus–Sorghum	25.3	21.0	21.5	17.8	18.0	y = 23.62 − 0.015x	0.78	0.06
CW1 (cm)	Cactus–pigeon pea	21.3	19.9	31.0	19.3	21.3	y = 21.36 + 0.031x − 7.67e^−5^x^2^	0.15	0.44
	Cactus–Sorghum	22.5	19.6	21.1	18.8	20.0	y = 22.14 − 0.020x + 3.26e^−5^x^2^	0.61	0.81
CW2 (cm)	Cactus–pigeon pea	18.1	18.8	17.3	15.3	16.8	y = 18.25 − 5.27e^−3^x	0.50	0.39
	Cactus–Sorghum	16.1	18.0	16.5	15.0	15.1	y = 17.07 − 4.71e^−3^x	0.49	0.72
CW3 (cm)	Cactus–pigeon pea	20.5	18.5	19.6	16.5	19.5	y = 20.62−0.023x + 4.48e^−5^x^2^	0.57	0.74
	Cactus–Sorghum	14.4	18.1	12.0	12.1	10.0	y = 15.86 − 0.013x	0.57	0.49
CWM (cm)	Cactus–pigeon pea	27.1	28.0	24.3	26.0	22.3	y = 27.52 − 0.010x	0.64	0.31
	Cactus–Sorghum	27.0	28.8	26.8	28.3	24.3	y = 27.06 + 0.014x − 4.41e^−5^x^2^	0.69	0.53
CT1 (cm)	Cactus–pigeon pea	20.6	26.5	34.8	23.8	40.0	y = 23.26 + 0.030x	0.46	0.30
	Cactus–Sorghum	21.8	25.0	19.5	18.0	25.8	y = 23.98 − 0.043x + 1.01e^−4^x^2^	0.48	0.60
CT2 (cm)	Cactus–pigeon pea	15.6	16.0	13.5	20.0	17.0	y = 15.14 + 6.59e^−3^x	0.25	0.39
	Cactus–Sorghum	13.0	15.8	13.5	14.0	16.0	y = 13.68 + 3.97e^−3^x	0.28	0.57
CT3 (cm)	Cactus–pigeon pea	12.0	13.3	12.8	13.5	12.3	y = 12.12 + 0.011x − 2.35e^−5^x^2^	0.70	0.94
	Cactus–Sorghum	5.3	11.3	6.0	8.8	5.8	y = 6.53 + 0.021x − 5.11e^−5^x^2^	0.23	0.32
CTM (cm)	Cactus–pigeon pea	31.8	32.3	30.5	32.0	35.5	y = 32.17−0.015x + 5.02e^−5^x^2^	0.90	0.97
	Cactus–Sorghum	43.8	45.3	45.0	44.3	46.0	y = 44.27 + 2.99e^−3^x	0.38	0.99
CP1 (cm)	Cactus–pigeon pea	75.3	59.5	74.0	74.0	78.3	y = 68.34 + 0.020x	0.24	0.05
	Cactus–Sorghum	78.5	85.5	76.3	78.0	73.5	y = 81.44 − 0.016x	0.41	0.63
CP2 (cm)	Cactus–pigeon pea	69.3	69.0	61.0	62.3	65.3	y = 70.26 − 0.064x + 1.17e^−4^x^2^	0.74	0.37
	Cactus–Sorghum	58.8	75.3	65.3	69.0	64.5	y = 62.56 + 0.068x − 1.46e^−4^x^2^	0.30	0.47
CP3 (cm)	Cactus–pigeon pea	75.0	62.5	74.0	55.3	74.0	y = 75.10 − 0.11x + 2.35e^−4^x^2^	0.37	0.44
	Cactus–Sorghum	49.5	63.5	41.0	42.5	32.8	y = 55.27 − 0.048x	0.57	0.39
CPM (cm)	Cactus–pigeon pea	51.3	55.3	45.5	55.0	49.3	y = 51.75 − 2.59e^−3^x	0.01	0.76
	Cactus–Sorghum	61.3	57.5	54.0	50.3	45.3	y = 60.31 − 0.034x	0.98	0.13
CA1 (cm^2^)	Cactus–pigeon pea	419.5	385.7	670.9	433.8	446.0	y = 410.62 + 1.10x − 2.4e^−3^x^2^	0.23	0.38
	Cactus–Sorghum	491.2	465.2	471.7	425.4	423.8	y = 485.49 − 0.15x	0.87	0.96
CA2 (cm^2^)	Cactus–pigeon pea	344.1	420.4	329.4	303.1	301.4	y = 375.05 − 0.18x	0.46	0.25
	Cactus–Sorghum	273.4	381.2	324.6	316.9	324.9	y = 304.80 + 0.31x − 6.38e^−4^x^2^	0.15	0.80
CA3 (cm^2^)	Cactus–pigeon pea	423.4	360.3	386.5	298.5	407.6	y = 428.11 − 0.82x + 1.67e^−3^x^2^	0.64	0.82
	Cactus–Sorghum	194.1	232.9	187.8	184.6	122.3	y = 219.73 − 0.18x	0.68	0.81
MCA (cm^2^)	Cactus–pigeon pea	410.3	416.9	304.3	360.5	299.1	y = 402.45 − 0.23x	0.53	0.65
	Cactus–Sorghum	479.9	424.9	400.7	353.1	311.7	y = 462.86 − 0.35x	0.97	0.24
DMC	Cactus–pigeon pea	0.1	0.1	0.1	0.1	0.1	y = 0.060 + 1.4e^−5^x	0.25	0.45
	Cactus–Sorghum	0.1	0.1	0.1	0.1	0.1	y = 0.072 + 2.34e^−5^x	0.25	0.63

PH (plant height), PW (plant width), and DMC (dry matter content) were evaluated. The remaining variables refer to cladode measurements: Length (CL), Width (CW), Thickness (CT), Perimeter (CP), and Area (CA). The suffixes 1, 2, and 3 indicate, respectively, the first, second, and third order of the cladodes, and the suffix M (or MCA, for area) denotes the mean of these measurements.

## Data Availability

The original contributions presented in this study are included in the article. Further inquiries can be directed to the corresponding authors.
